# Experimental study on impermeability of Loess liner mixed with bentonite-HDTMA

**DOI:** 10.1038/s41598-023-35433-9

**Published:** 2023-05-30

**Authors:** Zhang Ming, Hu Dongke, Pan Shaoyu, Chen Guozhou

**Affiliations:** 1 Zhengzhou University of Aeronautics, No. 15 Wenyuan West Road, Zhengdong New District, Zhengzhou, 450006 China; 2 Northwest University, Xi’an, 710127 China; 3 Henan Urban Planning Institute and Corporation, Zhengzhou, 450006 China

**Keywords:** Environmental sciences, Hydrology, Solid Earth sciences

## Abstract

Permeability tests are performed by using the flexible wall permeameter to study the influence of bentonite-HDTMA (hexadecyl trimethyl ammonium bromide) on the permeability performance of Loess as a lining material in the solid waste landfill. Results show, the impermeability of compacted Loess in the middle and lower reaches of the Yellow River in China does not meet the standard requirement as the landfill liner. The permeability of Loess mixed with more than 10% bentonite ratio is less than 1.0 × 10^−7^ cm·s^−1^. The permeability of Loess increases slightly after mixing a small amount of HDTMA. The impermeability of Loess mixed with some bentonite-HDTMA ratio still meet the standard requirement. The HDTMA may destroy the soil aggregate structure and increase the soil permeate channel. The SEMs show that the bentonite clay particle can fill the pores between Loess coarse particles and improve the impermeability performance of the material. The digital photos show that HDTMA can effectively resist the development of soil macro-cracks induced by wetting–drying cycles, better for liner to maintain good impermeability. On this basis, the relationship between the hydraulic conductivity of bentonite-HDTMA modified Loess and dry density is constructed. From this study, Loess can be used as a lining material for landfills, when mixed with bentonite/HDTMA at ratios of 10%/0% or 14%/2%.

## Introduction

Sanitary landfill is one of the final disposal methods for solid waste (general industrial solid waste and domestic waste). Landfill needs to be lined for intercepting pollutants, including heavy metals and organic compounds, from living environment. Traditionally, the clay is used as lining material for the waste disposal^1,2^. The Loess is widely distributed in various regions such as the middle and lower reaches of the Yellow River in China, the great plains and central lowland along the Missouri-Mississippi River in America, and the areas near the foothills and lower mountain belt of the Alps and the Carpathian Mountains in Europe, where clay is lacking at the same time. However, the hydraulic conductivity of Loess after compaction may exceeds the upper limit specified in the standard^1,3^.

The permeability of Loess is influenced by various factors such as dry density, ion concentration, temperature, and so on. Samples with dry density of 1.45 g·cm^−3^ or higher experience pore enlargement during seepage due to chemical reactions^4,5^. The permeability of Loess is sensitive to the concentrations of CaCl_2_ solutions. The concentration of CaCl_2_ affects particle flocculation and pore structure development, causing particle disintegratio^6^. The permeability increasing with temperature at 10 °C and 20 °C, but decreasing at 30 °C^5^. And the test results show the permeability of Loess is more than 1.0 × 10^−7^ cm·s^−1^.

Studies have explored the use of bentonite modified Loess as a lining material for landfills. Zhang et al.^7^ and Xi^8^ mixed the Loess in the middle and lower reaches of the Yellow River with 14% and 4% bentonite ratios, and the hydraulic conductivity of the modified loess is lower than or in the order of 1.0 × 10^−7^ cm·s^−1^. Liu et al.^9^ mix 6% to 7% bentonite into the Loess in North China, and the hydraulic conductivity of the mixture is 9.0 × 10^−8^ cm·s^−1^. Most of the test results show that the hydraulic conductivity of modified soil in other countries is less than 10^−7^ cm·s^−1^ when the bentonite ratio is around 15%^10–12^. That is, Loess modified by bentonite can meet the impermeability requirement.

**Figure 1:**
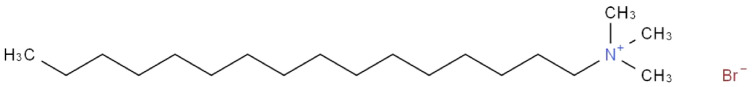
HDTMA chemical structure.

However, pure Loess or bentonite modified Loess has limited adsorption capacity for certain pollutants. The batch test shows that HDTMA (Hexadecy ltrimethyl Ammonium Bromide) can significantly improve the adsorption performance of some heavy metals and organic pollutants^13–15^, its chemical structure is shown in Fig. 1. HDTMA is a cationic surfactant, which is positively charged when dissolved in water. HDTMA can be adsorbed on the surface of clay mineral and react with pollutant through exchange adsorption and non-exchange adsorption^16,17^, so as to achieve the effect of adsorbing or immobilising pollutant. From the perspective of environmental science, it is possible to use bentonite-HDTMA modified Loess as a lining material for solid waste landfill. Meanwhile, the phenomenon of wetting–drying cycle in the liner happen commonly, which may lead to the increasement of crack related to the impermeability performance^18^.

This study aims to investigate the effect of bentonite and HDTMA on the impermeability of Loess by using the flexible wall permeameter for the first time, focus on the effect of HDTMA on the impermeability of Loess after wetting–drying cycles. The goal is to provide essential research data for the localization research of lining material for the solid waste landfill in the Loess area.

## Materials and methods

### Materials

**Figure 2:**
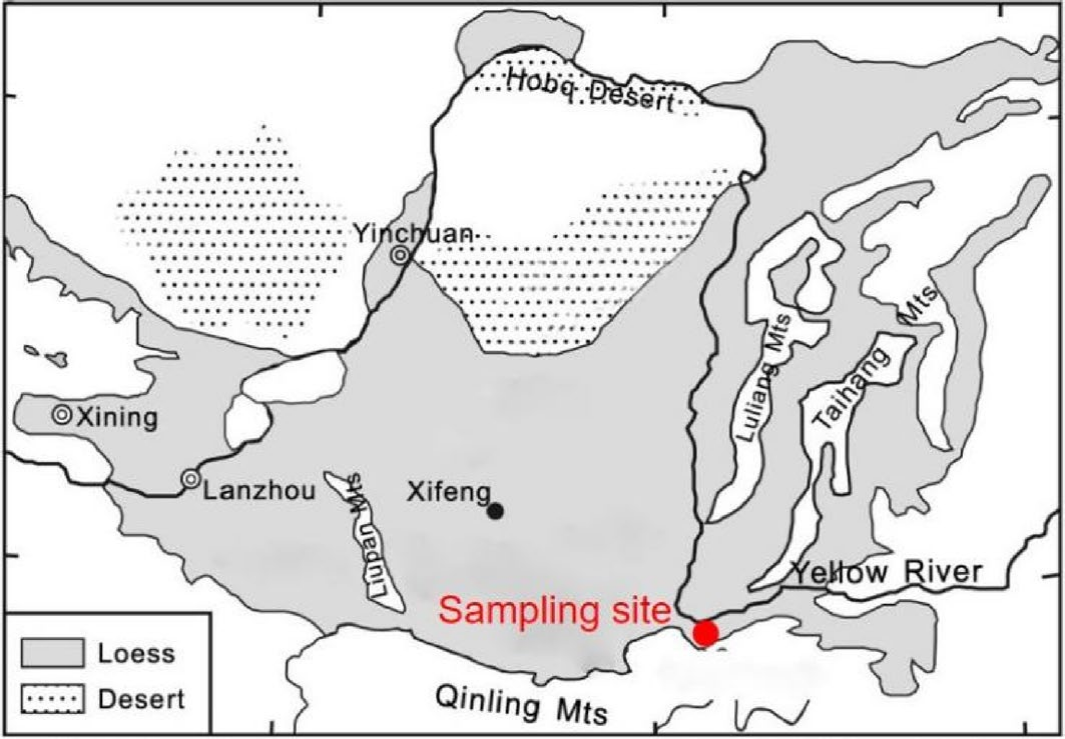
Schematic diagram of sampling site.

The material used in the test is Malan Loess in the middle and lower reaches of the Yellow River in China (Fig. 2). The topsoil in the site is usually removed before setting the lining layer in engineering practice. Therefore, the sampling depth of this study is 1.0–4.0 m. Undisturbed sample is characterized by a high content of fine particle and plasticity index of 8.5, which can be considered as a typical silt. The bentonite used in the study is purchased from Shandong province close to the site. The basic physical properties of Loess and bentonite are shown in Table 1. The tests for particle percentage, specific gravity, liquid limit, and Plastic limit follow standard for geotechnical testing method of China^19^. The HDTMA production is purchased from the commercial company, its purity is 99%.

**Table 1:** Basic physical properties of materials in the test.

Sample	Particle percentage (%)					Specific gravity	Liquid limit (%)	Plastic limit (%)	Type
	0.25–0.075 mm	0.075–0.05 mm	0.05–0.01 mm	0.01–0.005 mm	< 0.005 mm				
Loess	1.9	38.5	31.1	5.7	22.8	2.70	25.9	17.4	Silt
Bentonite	2.4	16.1	17.8	13.3	50.4	2.76	78.3	41.3	Clay

The bentonite with mass ratio of 0–22% and HDTMA with 0–4% are mixed into the Loess, respectively. The material is stir evenly, then the distilled water is spray according to the predetermined water content for making the material evenly humidified, finally place them in the moisturiser for 60 h. The compaction test results show that the optimum water content of the modified Loess is about 18.0%, and the maximum dry density^20^ is about 1.70 cm·s^−1^. In order to better control the dry density of the samples, the variable compaction energy static compaction method is used to sampling in this study^21,22^. The instrument is a TYA-3000 electro-hydraulic pressure testing machine. This research considers three factors, that are, bentonite ratio (*R*(Bentonite)), HDTMA ratio (*R*(HDTMA)) and dry density (*ρ*). Referring to the previous test results of the hydraulic conductivity of bentonite modified Loess^23^, the basic parameters of the samples in this test are shown in Table 2. After compacted, the SEM photos for samples are obtained by JSM-7001F instrument.

**Table 2:** The basic parameters of samples.

No.	Bentonite ratio (%)	HDTMA ratio (%)	Water content (%)	Specific gravity	Dry density (g·cm^−3^)
M1	0	0	18.5	2.70	1.70
M2	6	0	18.2	2.70	1.69
M3	6	2	17.1	2.70	1.68
M4	10	0	17.4	2.70	1.69
M5	10	2	19.3	2.71	1.36
M6	10	2	19.3	2.71	1.57
M7	10	2	17.8	2.71	1.69
M8	10	4	17.5	2.71	1.67
M9	14	0	18.3	2.71	1.69
M10	14	2	18.4	2.71	1.68
M11	100	0	20.8	2.74	1.60
M12	18	0	18.6	2.71	1.69
M13	22	0	18.5	2.72	1.69

### Methods

**Figure 3:**
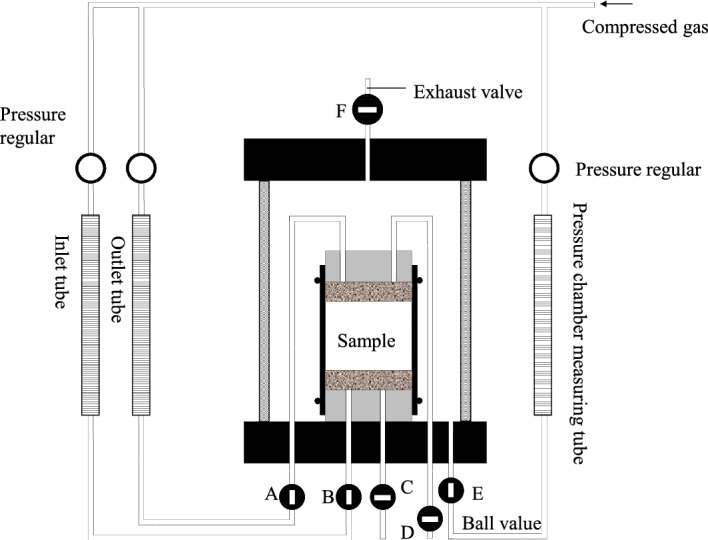
Schematic diagram of flexible wall permeameter.

The permeability test in this study is performed by using the flexible wall permeameter (show in Fig. 3). The permeation test was carried out at room temperature of 20 °C, with distilled water as the permeation liquid. Compared with traditional rigid wall permeameter, the flexible wall permeameter in this test can effectively avoid sidewall leakage by applying back pressure, shorten the test time, accurately control the principal stress state, finally improving the test accuracy and precision. According to the ASTM D 5084-16a^24^, the sample is located into the flexible wall permeameter, with the permeable stone and filter paper placed in sequence on the top and bottom of the sample.

Due to the extremely low permeability, it is difficult in saturating the sample. The back pressure saturation is used firstly in this test (Fig. 3): (1) After installing the sample in the permeation chamber, open the ball valve F and the ball valve E, and fill the permeation chamber with water. When the water is full, close valve F. A certain confining pressure is applied to the sample in the permeation chamber by adjusting the pressure regulator connected to the measuring tube of the pressure chamber. (2) Fill both the inlet and outlet tubes with water, open ball valves A and B, and close C and D. Adjust the two pressure regulators connected to the inlet and outlet tube to equalize the pressure. The compressed gas exerts a certain back pressure on the sample through the inlet and outlet tube. (3) The real-time monitoring of changes in water levels in the inlet and outlet tubes is performed. If the height of the water head of the two measuring pipes are equal and there is no obvious change, the sample can be considered to be saturated and the back pressure saturation process is terminated.

After the back pressure saturation process, the permeation test was carried out. During the test, the infiltration flow was monitored through the water inlet tube, and the hydraulic conductivity of the sample was calculated according to the variable head method. The criteria for termination of the permeability test includes: (1) Leachate from the water outlet tube, means permeating through the sample, at least equal to one pore volume of the sample, (2) the flow rate for water inlet is equal to the flow rate for water outlet, (3) the hydraulic conductivity remains stable. The three above requirements have been met in this test, so the value before the termination of the test is selected as the final hydraulic conductivity value of the sample. Then the osmotic pressure and confining pressure should be released step by step according to the method of decreasing 100 kPa every 30 min to avoid unpredictable deformation of the sample caused by sudden unloading during sample removal.

The formula for the hydraulic conductivity using by using the flexible wall permeameter is:

1 $$ K = 2.3\frac{al}{{At}}\lg \frac{h1}{{h2}} $$

where: *K* is the permeability coefficient, *a* is the cross-sectional area of the tube (cm^2^), *l* is the length of the sample (cm), *A* is the cross-sectional area of sample (cm^2^), t is permeability time (s), *h*1 is Initial water head (cm), *h*2 is ending water head (cm).

After the permeation test, all samples are put into the containers with rigid wall separately, then perform evaporative drying (not oven drying)-confined saturation (not vacuum saturation) cycle test in a closed environment, where is windless with a constant temperature of 20 ± 2 °C. The water content of the sample does not change after three days of continuous measurement, meaning one wetting–drying cycle finish (about 15 days), totally experience 5 cycles for each sample. At the end of each cycle, the digital photos are took to observe the changing in apparent cracks. Finally, the hydraulic conductivity of M9 and M10 is measured once again after undergoing 5 cycles of wetting and drying, to research the influence of HDTMA on the impermeability performance of modified Loess under wetting and drying cycles.

## Results

### Hydraulic conductivity of bentonite-HDTMA modified Loess

**Figure 4:**
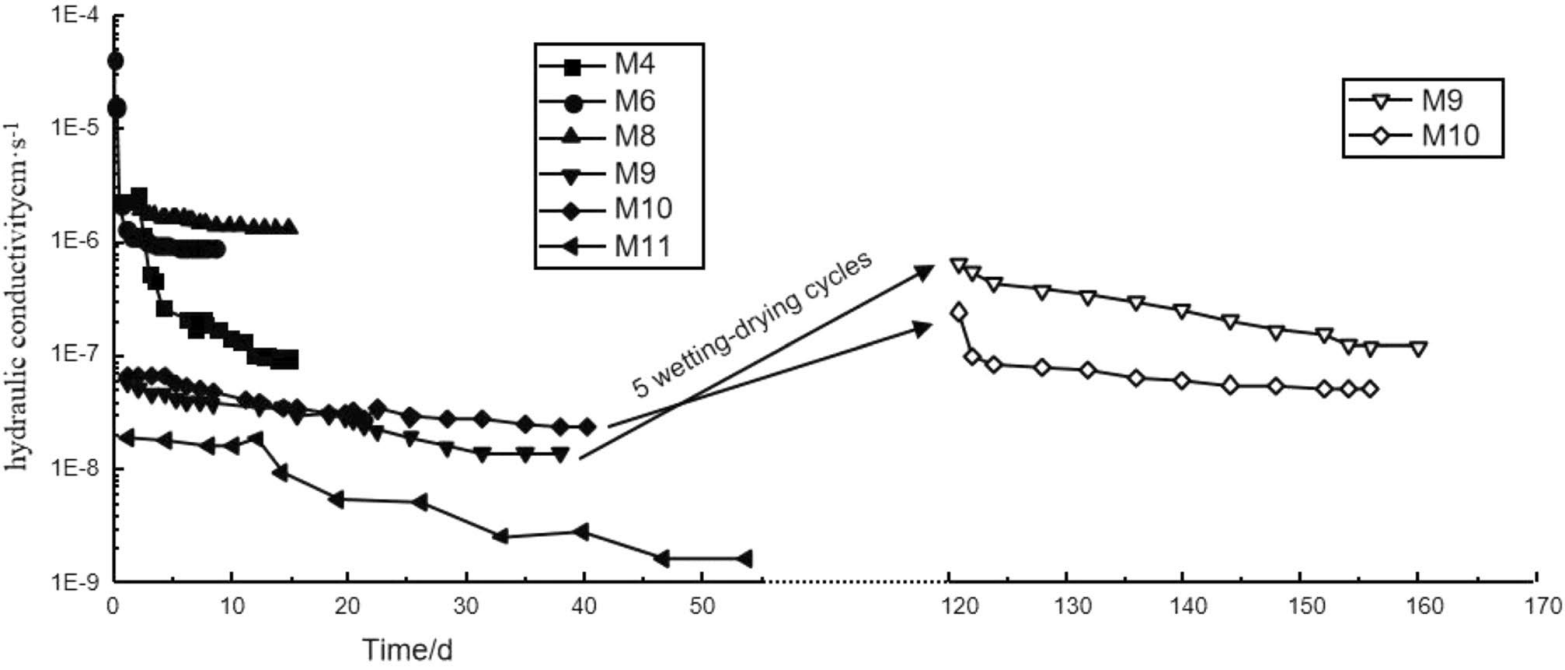
Hydraulic conductivity of bentonite-HDTMA modified Loess vs. Time.

The permeability tests were carried out on Loess mixed with different HDTMA and bentonite ratio. Figure 4 shows the trend of the hydraulic conductivity of bentonite-HDTMA modified Loess with time. Actually, there are total 15 groups of permeability tests (including two groups after 5 wetting–drying cycles), all the curves are chaotic when plotted them on the one graph. In this section, just six typical curves of M4, M6, M8, M9, M10 and M11 are used to analyze the changing trend of hydraulic conductivity with time. The time for both permeability test and wetting–drying cycles last 160 days, as shown in Fig. 4 and Table 3. It can be seen from the figure that the hydraulic conductivity decreases with the penetration time increasing, and finally tends to be stable. In the early stage of penetration, the hydraulic conductivity decreases significantly, which can exceed one order of magnitude. In the later stage of penetration, the hydraulic conductivity basically remains unchanged and reaches a stable value. Including the samples after 5 wetting–drying cycles, the permeability tests of all samples show the same trend. The more bentonite ratio in the modified Loess, the longer time needed for permeation stability. The longest time for the permeation stability of sample is pure bentonite, that is 54 days.

**Table 3:** The hydraulic conductivity of samples.

No.	Time (day)	Hydraulic conductivity (cm·s^−1^)
M1	6.27	1.30E−7
M2	13.05	3.43E−7
M3	6.55	4.05E−7
M4	14.95	9.52E−8
M5	6.55	2.08E−6
M6	8.55	8.97E−7
M7	10.05	1.72E−7
M8	14.85	1.32E−6
M9	38.02	1.40E−8
M10	40.17	2.40E−8
M11	53.78	1.67E−9
M12	58.02	4.80E−9
M13	60.85	2.20E−9

Test results show that, the hydraulic conductivity of samples M1–M13 ranges from 1.15 × 10^−9^ to 2.08 × 10^−6^ cm·s^−1^. However, the values of M9 and M10 are 1.21 × 10^−7^ and 5.20 × 10^−8^ cm·s^−1^ after 5 wetting–drying cycles, respectively. That means, the hydraulic conductivity of Loess modified by bentonite simply can no longer meet the standard requirement after 5 wetting–drying cycles. Additionally mixing some HDTMA, the hydraulic conductivity of the modified Loess increases slightly, but remains 10^−8^ cm·s^−1^ magnitude, meeting the standard requirement in China. In a word, the bentonite and HDTMA have an obvious effect on the hydraulic conductivity of Loess. The hydraulic conductivity of the lining material required by the China standard does not exceed 1.00 × 10^−7^ cm·s^−1^. Therefore, it is particularly critical to determine the bentonite and HDTMA ratio in the modified Loess.

### Cracks on the surface of bentonite-HDTMA modified Loess after wetting–drying cycles

**Figure 5:**
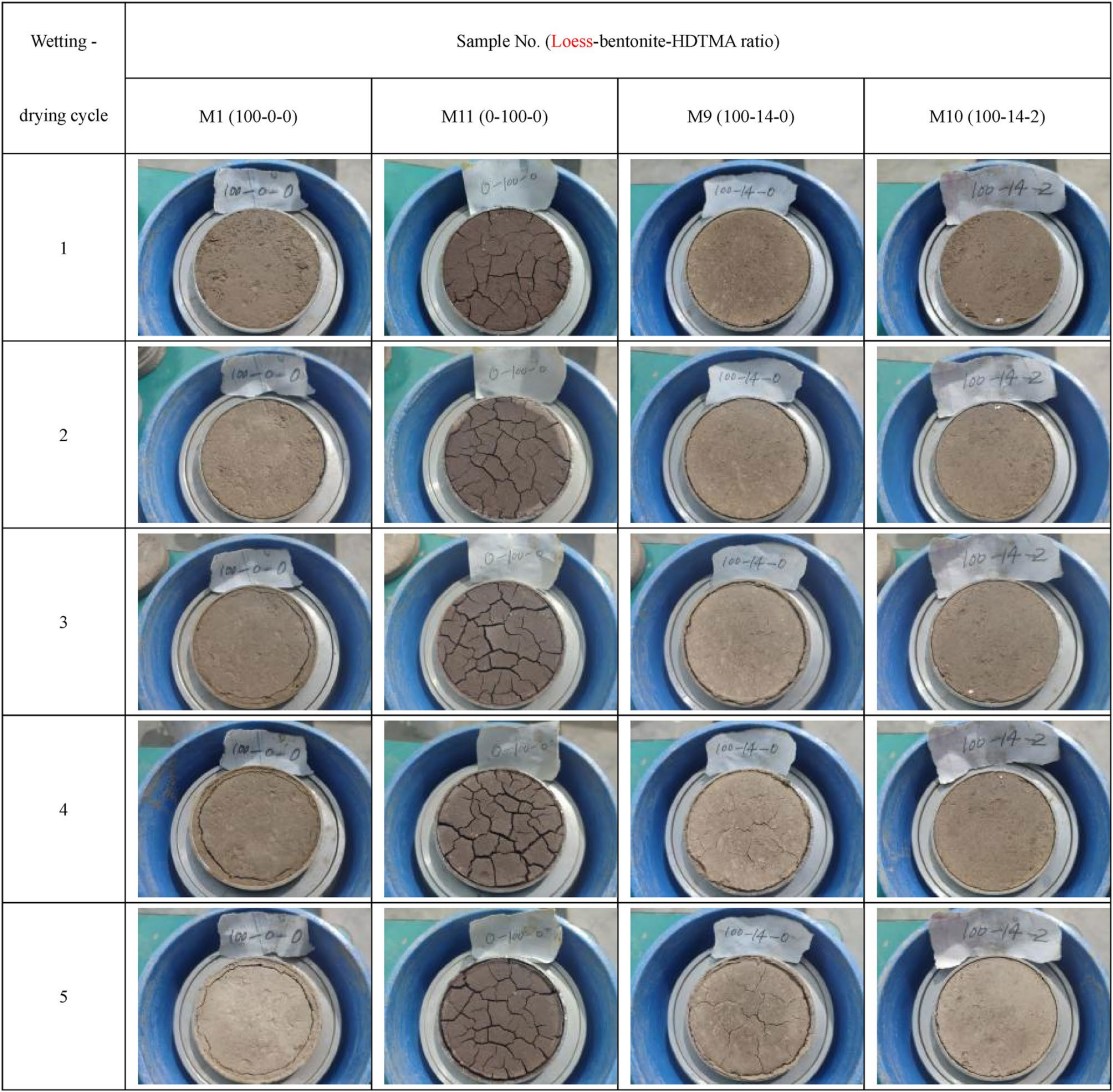
Cracks on the surface of bentonite-HDTMA modified Loess after wetting–drying cycles.

Figure 5 shows the apparent crack characteristics of the modified Loess after 1 to 5 wetting–drying cycles. After 5 wetting–drying cycles, the pure bentonite sample (M11) develops the most significant crack: the cracks start from the around of the sample to the center, expand to a certain distance and then branch and expand again. The cracks of bentonite modified Loess sample (M9) develop secondly. The cracks of Loess samples (M1) and bentonite-HDTMA modified Loess samples (M10) hardly develop and the samples only experience volume shrinkage. In addition, the development of cracks is gradually obvious as the wetting–drying cycles increasing for all samples. The bentonite modified Loess without HDTMA will develop crack after wetting–drying cycles, the crack develop severely with the times of wetting–drying cycle increasing. The solid waste landfills often undergo wetting and drying cycles in the both dry and rainy seasons during the engineering practice. By adding a small amount of HDTMA, the degradation effects of these wetting and drying cycles on the liner's impermeability performance can be reduced.

## Discussion

### Effect of bentonite ratio on the hydraulic conductivity of modified Loess

**Figure 6:**
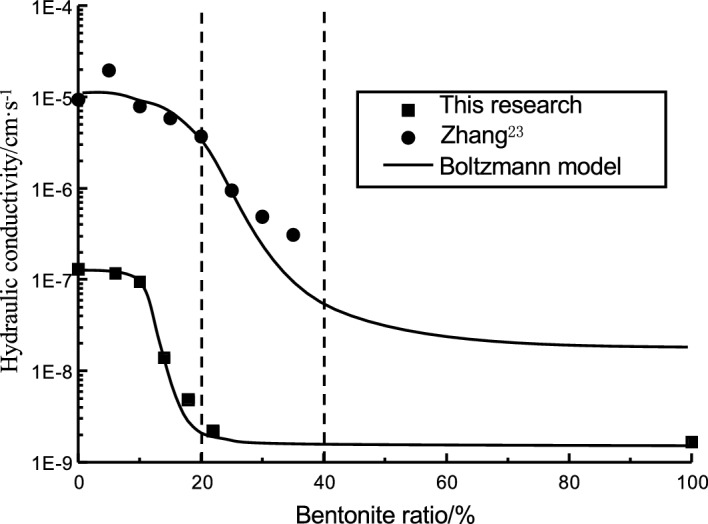
Hydraulic conductivity of bentonite-HDTMA modified Loess vs. Bentonite ratio (*R* (HDTMA) = 2%).

Figure 6 shows the relationship between the hydraulic conductivity of bentonite-HDTMA modified Loess and the bentonite ratio, including research results of Zhang^23^ sampling from the northwest region of China. It is considered that the hydraulic conductivity of Loess mix with small bentonite ratio should be close to that of the pure bentonite. The hydraulic conductivity of soil decreases slowly. When the bentonite ratio increase to a certain value, the hydraulic conductivity of modified Loess decreases at a certain rate. As the bentonite ratio continues to increase, the decreasing rate of hydraulic conductivity gradually slowed down, and finally tend to a certain stable value. The hydraulic conductivity of modified Loess is close to pure Loess when the bentonite ratio is small. On the contrary, the hydraulic conductivity of modified Loess is close to pure bentonite when the bentonite ratio is large. The test results of Chapuis et al.^25–27^ also show the same trend, even the coarse-grained soil is different from this research.

Based on the above analysis, the hydraulic conductivity of bentonite modified Losses decreases in an inverse S-shaped with the bentonite ratio increase, which fit the classical Boltzmann model. So the Boltzmann model is used for fitting the test data, including the test results of Zhang^23^. The bentonite ratios of the modified Loess in this test are 0%, 6%, 10%, 14%, 18%, 22% and 100%, respectively. The curve sharp for the two tests is similar in form. Because the hydraulic conductivity value of pure Loess in this test (1.30 × 10^−7^ cm·s^−1^) is less than that of Zhang (9.34 × 10^−6^ cm·s^−1^), the fitting curve of this test overall downward to Zhang^23^. This also means that the bentonite ratio for the modified Loess in the middle and lower reaches of the Yellow River can be less than that in the northwest region of China, in order to obtain the same impermeability performance. According to the mathematical expression of Boltzmann model, the relationship between the hydraulic conductivity of modified Loess and the bentonite ratio can be expressed as:

2 $$ \frac{{{\text{lg}}k - {\text{lg}}k\;(Bentonite)}}{{{\text{lg}}k\;(Loess) - {\text{lg}}k\;(Bentonite)}} = \frac{1}{{1 + {\text{exp}}\left[ {\left( {R\;(Bentonite) - R\;(Bentonite0)} \right)/d} \right]}} $$

After arranging formula (2),

3 $$ \lg k = \lg k\;(Bentonite) + \frac{\lg k\;(Sand) - \lg k\;(Bentonite)}{{1 + \exp \left[ {\left( {R\;(Bentonite) - R\;(Bentonite0)} \right)/d} \right]}} $$

where, *k* (Bentonite) is the hydraulic conductivity of pure bentonite, cm·s^−1^; *k* (Loess) is the hydraulic conductivity of pure Loess, cm·s^−1^; *R* (Bentonite0) is the bentonite ratio when *k* = (*k* (Bentonite) + *k* (Loess))/2, % , could be called "half-life ratio"; *d* is the fitting parameter.

It can be seen from the fitted curve (Fig. 6) and Eq. (3) that, the bentonite ratio should be more than 10% when the hydraulic conductivity is less than 1.0 × 10^−7^ cm·s^−1^. And the hydraulic conductivity decreases not so obvious when the bentonite ratio is more than 20%. From an economical point of view, it is recommended that the bentonite ratio ranges from 10 to 20%.

### Effect of HDTMA on the hydraulic conductivity of modified Loess

**Figure 7:**
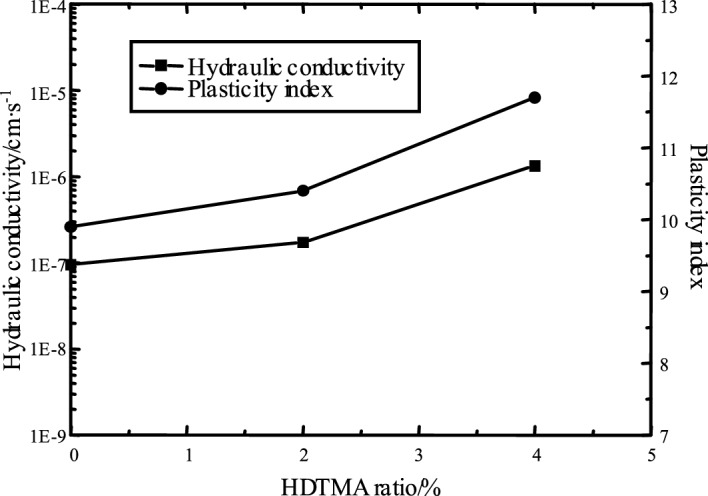
Hydraulic conductivity of bentonite-HDTMA modified Loess by vs. HDTMA ratio (*R* (Bentonite) = 10%).

Figure 7 shows the relationship between the hydraulic conductivity of bentonite-HDTMA modified Loess and the HDTMA ratio. As shown in Fig. 7, the hydraulic conductivity of modified Loess increased from 9.52 × 10^−8^ to 1.32 × 10^−6^ cm·s^−1^ with the HDTMA ratio increasing from 0 to 4%. Overall, the hydraulic conductivity of the modified Loess is increased after mixed with HDTMA, and the impermeability performance is weakened. This is because the HDTMA can significantly reduce the surface tension between the interfaces of different phases, the bentonite fine particles aggregated around the Loess coarse particles easily disperse into the water phase until the colloidal state appears in the saturated state^28^. The inherent aggregate structure of the soil is destroyed, and the soil particle is dispersed. This can be reflected from the relationship between the plasticity index of modified Loess and the HDTMA ratio (Fig. 7): the plasticity index of modified Loess increases with the HDTMA ratio increasing. In addition, the strong interaction forces (mainly including hydrophobic forces and van der Waals forces) are generated between HDTMA and soil particles^29^, which can further change the pore structure characteristics of soil particles, resulting in an increase for the permeability. the result shows that the HDTMA has a positive regulating effect on the water-physical property of modified Loess, but has the effect on the increase of hydraulic conductivity without considering the wetting–drying cycle. The hydraulic conductivity of modified Loess remains in the order of 10^−7^ after mixing 2% HDTMA.

### Effect of dry density on the hydraulic conductivity of modified Loess

**Figure 8:**
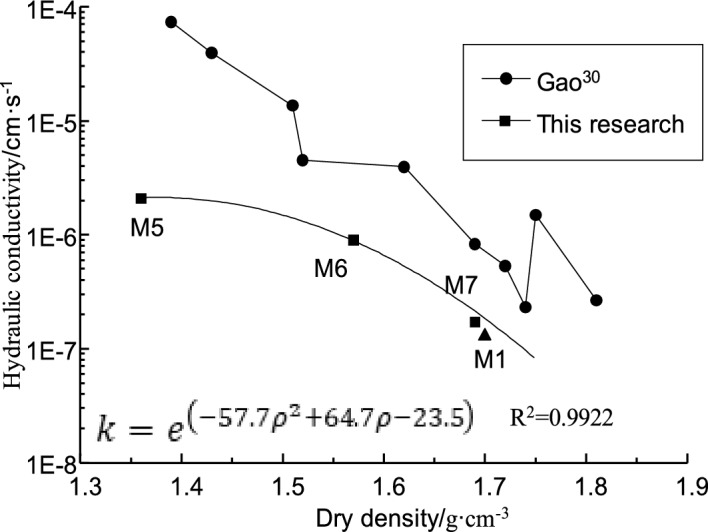
Hydraulic conductivity of bentonite-HDTMA modified Loess vs. Dry density.

Figure 8 shows the relationship between the hydraulic conductivity of bentonite-HDTMA modified Loess and dry density. As shown in Fig. 8, the hydraulic conductivity of modified Loess reduces from 2.08 × 10^−6^ to 1.72 × 10^−7^ cm·s^−1^ with the dry density increasing from 1.36 g·cm^−3^ (M5) to 1.69 g·cm^−3^ (M7). It is easy to understand, the porosity of the sample decreases as the dry density increasing, and the water permeate channel decreases, ultimately leading to the decrease of the hydraulic conductivity and the enhancement of the impermeability performance.

Previous studies^30,31^ have shown that the hydraulic conductivity of pure Loess decreases with the dry density increasing, and the decreasing trend gradually slows down. When the dry density of Loess is greater than 1.70 g·cm^−3^, the hydraulic conductivity is almost unchanged and keep a very low level even the dry density increasing (Fig. 8). According to the previous research^20^, the dry density for bentonite-HDTMA modified Loess after standard compaction is usually less than 1.70 g·cm^−3^. With the dry density increasing, the hydraulic conductivity of soil samples shows a nonlinear decreasing trend. After nonlinear regression analysis, the functional relationship between the hydraulic conductivity and dry density for Loess when the dry density is less than 1.70 g·cm^−3^ is shown in formula (4):

4 $$ k\;({\text{Loess}}) = e^{{({\text{a}}\rho_{{}}^{2} + {\text{b}}\rho - {\text{c}})}} $$

where, *ρ* is the dry density of Loess, g·cm^−3^; *a*, *b* and *c* are fitting parameters.

It can be seen from Fig. 8 that, although a small amount of HDTMA is mixed, the hydraulic conductivity of bentonite-HDTMA modified Loess also has an exponential relationship with the dry density, showing the same changing trend with pure Loess. The functional relationship fitted by formula (4) is shown in formula (5), which can be used as a rapid evaluation method for rapid evaluation of the impermeability performance of bentonite-HDTMA modified Loess in situ.

5 $$ k = e^{{( - 57.7\rho_{{}}^{2} + 64.7\rho - 23.5)}} $$

### Microstructure of bentonite-HDTMA modified Loess

**Figure 9:**
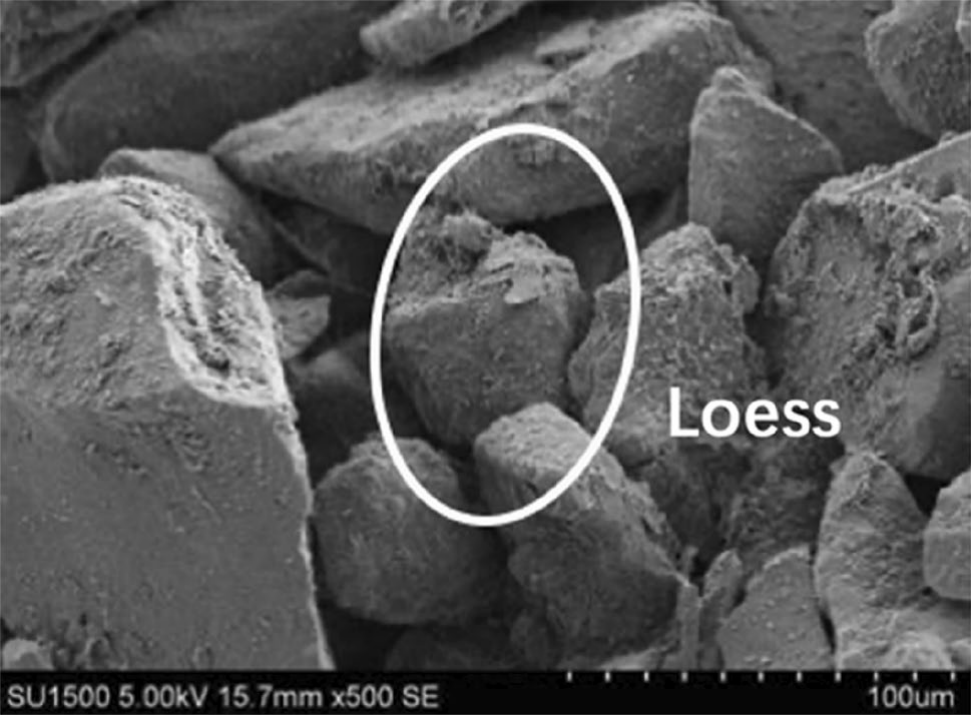
Yulin Loess^23^.

**Figure 10:**
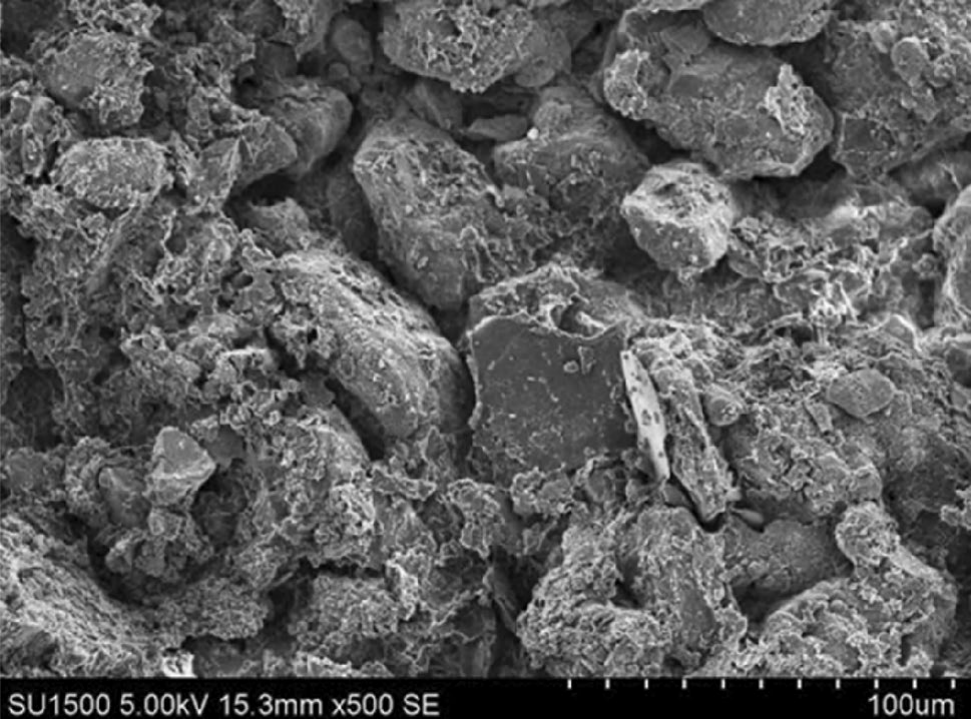
Sanmenxia Loess.

**Figure 11:**
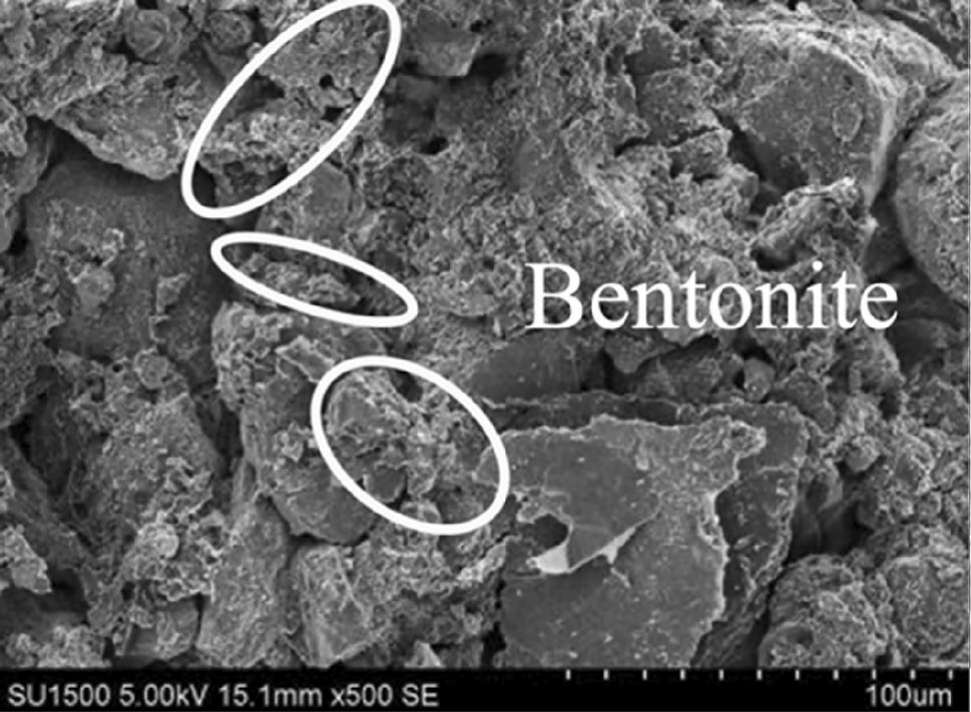
Bentonite modified Loess.

**Figure 12:**
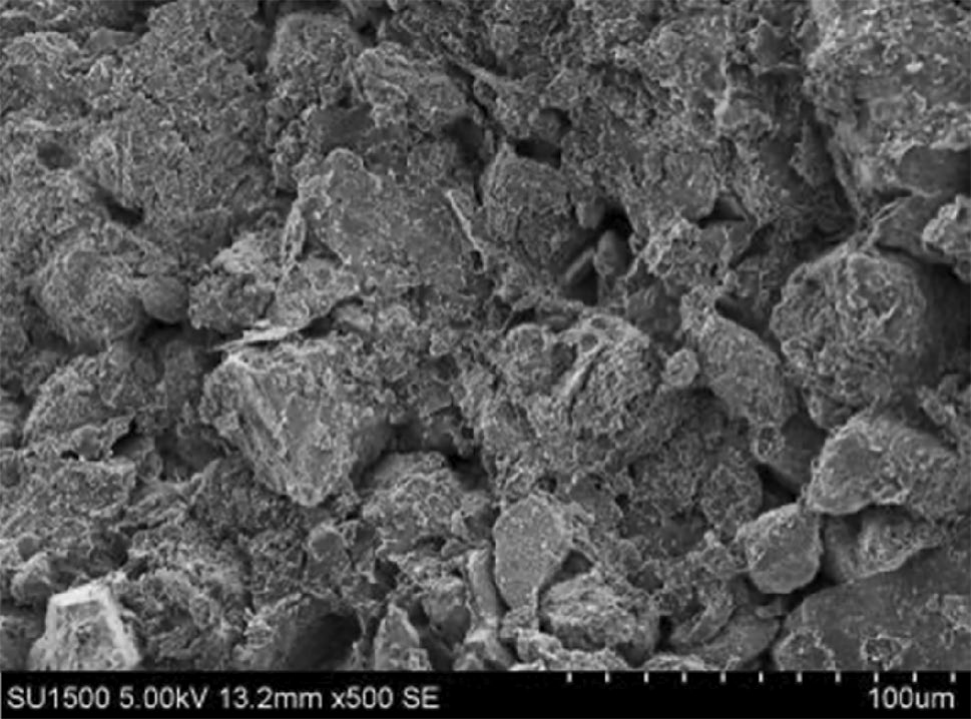
Bentonite-HDTMA modified Loess.

The clay fine particles should increase with the bentonite ratio increasing. The clay fine particle will fill into the pore of Loess coarse particles, reducing the permeate channel of soil, and finally enhance the impermeability performance. As shown in Figs. 9, 10, 11, 12, the pores of Loess coarse particles are fully filled with clay fine particles after mixing 10% bentonite (Fig. 11), and the permeate channel is significantly reduced, resulting in the hydraulic conductivity decreases sharply. According to the relationship between the hydraulic conductivity of modified Loess and bentonite ratio (Fig. 6), the coarse particles are expected to be “suspended” in the clay fine particles when the bentonite ratio exceeds 20%, there is no pores between Loess coarse particles for clay to fill in, the hydraulic conductivity will no longer decrease significantly. Combined with the test results of Yulin Loess^23^, the above point of view can be confirmed: the content of particles smaller than 0.005 mm in this test is as high as 22.8%, but the content of particles with the same size for the Yulin Loess is only 8.5%, as shown in Figs. 9 and 10. Due to the high content of clay fine particle, the Loess coarse particle in this test is the first to reach the "suspended" state, and less bentonite ratio is required to reach the stable value of hydraulic conductivity (in this test, *R* (*Bentonite*) = 20%; Zhang et al., *R* (*Bentonite*) = 40%), shown in Fig. 6.

In addition, as shown in Fig. 12, the clarity of SEM for the clay layered structure is weakened after mixing the HDTMA, which may be due to the coating of HDTMA organic long chains on the surface of clay particles, and the surface of soil particles has the trend from unevenness to smoothness. This indirectly proves that the HDTMA, as an organic cation, undergoes an exchange reaction with the exchangeable cations between the clay particle layers, then arranges between the clay layers in a single-layer or multi-molecular layer structure, thereby widens the clay layer spacing^32^. Consequently, the dry density of the modified Loess decreases and the impermeability performance is weakened.

**Figure 13:**
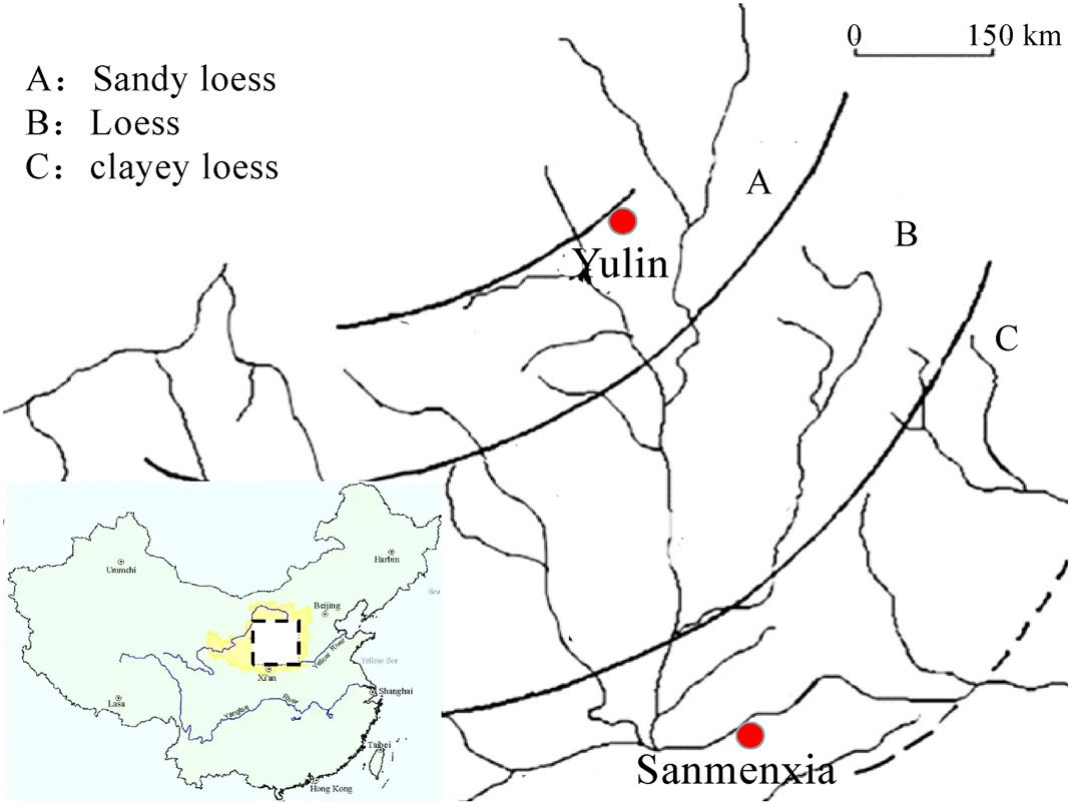
Distribution of 3 types of Loess in China^33^.

According to the particle size of the Loess, the Loess distribution in China can be divided into three regions: A, B, and C (see Fig. 13). It should be noted, the Loess in this test is taken from Sanmenxia, which belongs to clay Loess (Area C), the Yulin Loess test by Zhang et al.^23^ belongs to sandy Loess (Area A). The difference in particle size leads to a great difference in the control effect of bentonite ratio on the hydraulic conductivity for modified Loess. The Loess in this test contains more clay fine particle, and the permeability test also shows that the Loess in region C needs less bentonite ratio to meet hydraulic conductivity required in China standard than region B. It can be seen that, the bentonite modified Loess has better application prospect in the clayey Loess region, overlapping with the middle and lower reaches of the Yellow River.

## Conclusion

The Sanmenxia Loess, located in the middle and lower reaches of the Yellow River is selected, mixed with different bentonite and HDTMA ratio. The permeability test is carried out by using the flexible wall permeameter to explore the control effect of bentonite-HDTMA on the impermeability performance of Loess as lining material. The bentonite and HDTMA ratios for the liner are proposed, leading to the following conclusions:

1. the impermeability performance of pure Malan Loess did not meet China's standard requirements for lining materials in site. The addition of 10% to 20% bentonite effectively improve the impermeability performance of the Loess by filling the pores between coarse particles, and this improvement is not obvious when the bentonite ratio exceeded 20%. The relationship between the hydraulic conductivity of the modified Loess and the bentonite ratio follows the Boltzmann model.
2. The addition of HDTMA slightly increased the hydraulic conductivity of the modified Loess by disrupting the soil's inherent aggregate structure. It should be noted that, HDTMA can effectively reduced the deterioration effect of wetting–drying cycles on the impermeability performance of the modified Loess.
3. The hydraulic conductivity of the bentonite-HDTMA modified Loess has an exponential relationship with dry density. The study recommended a bentonite/HDTMA ratio of 10%/0% or 14%/2% for the modified Loess as a lining material in site, which are preliminary reference values for the optimal bentonite/HDTMA ratio in future research.

## Data Availability

The datasets used and analysed during the current study available from the corresponding author on reasonable request.
