# Author Correction: Experimental study on impermeability of Loess liner mixed with bentonite-HDTMA

**DOI:** 10.1038/s41598-023-37581-4

**Published:** 2023-06-27

**Authors:** Zhang Ming, Hu Dongke, Pan Shaoyu, Chen Guozhou

**Affiliations:** 1 Zhengzhou University of Aeronautics, No. 15 Wenyuan West Road, Zhengdong New District, Zhengzhou, 450006 China; 2 Northwest University, Xi’an, 710127 China; 3 Henan Urban Planning Institute and Corporation, Zhengzhou, 450006 China

Correction to: *Scientific Reports* 10.1038/s41598-023-35433-9, published online 30 May 2023

The original version of this Article contained an error in the Acknowledgements section.

“Thank you for the financial support from Henan Province Science and Technology Research Project (No. 212102310968), Key scientific research projects of colleges and universities in Henan Province (No. 21A410004), Henan Province Housing and Urban-Rural Construction Science and Technology Project (No. HNJS-2020-k25) and Post-doctoral research project of Henan Urban Planning Institute and Corporation.” now reads:

“Thank you for the financial support from Henan Province Science and Technology Research Project (No.222102320137), Key scientific research projects of colleges and universities in Henan Province (No. 21A410004), Henan Province Housing and Urban-Rural Construction Science and Technology Project (No. HNJS-2020-k25) and Post-doctoral research project (2022M721046)."

The original Article has been corrected.

